# Human resources for health in six healthcare arenas under stress: a qualitative study

**DOI:** 10.1186/s12960-015-0005-7

**Published:** 2015-03-29

**Authors:** Jo Durham, Enrico Pavignani, Mark Beesley, Peter S Hill

**Affiliations:** School of Public Health, The University of Queensland, Brisbane, Qld 4006 Australia

## Abstract

**Background:**

Research on “human resources for health” (HRH) typically focuses on the public health subsector, despite the World Health Organization’s inclusive definition to the contrary. This qualitative research examines the profile of HRH in six conflict-affected contexts where the public health subsector does not dominate healthcare service provision and HRH is a less coherent and cohesive entity: Afghanistan, the Central African Republic (CAR), the Democratic Republic of Congo (DR Congo), Haiti, the Occupied Palestinian Territories and Somalia.

**Methods:**

The study uses a multiple-country qualitative research design including documentary analysis and key informant interviews undertaken between 2010 and 2012. The documentary analysis included peer-reviewed articles, books, unpublished research and evaluations and donor and non-government organisation reviews. A common thematic guide, informed by this analysis, was used to undertake key informant interviews. Informants thought able to provide some insight into the research questions were identified from ministry of health organograms, and from listings of donors and non-government organisations. Local informants outside the familiar structures were also contacted. In CAR, 74 were interviewed; in Somalia 25; . in Haiti, 45; in Afghanistan, 41; in DR Congo, 32; and in the Occupied Palestinian Territories, 30. In addition, peer review was sought on the initial country reports.

**Results:**

The study discovered, in each healthcare arena investigated, a crowded HRH space with a wide range of public, private, formal and informal providers of varying levels of competence and a diverse richness of initiatives, shaped by the easy commodification of health and an unregulated market. The weak regulatory framework and capacity to regulate, combined with limited information regarding those not on the state payroll, allowed non-state providers to flourish, if not materially then at least numerically.

**Conclusion:**

When examining HRH, a reliance on information provided by the state health sector can only provide a partial and inadequate representation of reality. For policy-makers and planners in disrupted contexts to begin to appreciate fully current and potential HRH, there is a need to study the workforce using conceptual tools that reflect the situation on the ground, rather than idealised patterns generated by incomplete inventories and unrealistic standards.

## Background

This paper examines the profile of human resources for health (HRH) in six fragile and conflict-affected states, selected for the diversity of their social, political and historical evolution: Afghanistan, Central African Republic (CAR), Democratic Republic of Congo (DR Congo), Haiti, the Occupied Palestinian Territories and Somalia. It analyses the available data and the implications of a state-centric analysis where the reach of the state in the provision of health services is limited. It points to the reality of an active health workforce beyond the state: poorly quantified, largely unregulated and of variable quality—but nevertheless frequently the most numerous health service providers in these contexts. We suggest that the customary focus on HRH in the state health sector provides an inherently inaccurate picture. We argue, therefore, that in fragile and conflict-affected environments there is a need to adopt, in reality not just rhetoric, the World Health Organization’s inclusive definition of HRH—“all individuals engaged primarily in the improvement of the health of populations” [1], committing to the difficult task of tallying, or at least seriously estimating, the HRH actually available to the population.

### The health workforce

Modern health systems are pluralistic and often transnational in nature, making contemporary HRH a rich and complex system composed of public, private and informal operators [2]. The World Health Organization (WHO) inclusively defines health systems as “all the activities whose primary purpose is to promote, restore or maintain health” [3] and in doing so explicitly recognises that the health system extends beyond the public health realm. But the common understandings of the health system underestimate the size of the health workforce. It encompasses not just the clinical staff in the formal arena—medical, nursing, midwifery and other professionals—but also their managers and administrative staff. It includes non-professional healthcare providers, such as community health workers and vaccinators, and all other employees (such as auxiliary staff, even watchmen) who work not just in the public subsector but also that of the private and not-for-profit subsectors. Those in the informal health subsector must also be considered [4-6]. Strictly speaking, the WHO definition suggests an intention that would exclude the many providers whose aim is just to make a living out of health care—but who do contribute to some form of health service provision. But under this definition, market vendors of medicines and traditional practitioners, the sole healthcare providers in many rural localities—and the still-preferred choice in some urban ones—are a part of the HRH.

Most analyses default to an examination of the role of publicly employed HRH, where the bulk of data is available. To compound matters, such official data are regularly poor, to the point that even the public portion of the health workforce is misrepresented [6]. But as we show in this paper, exploring HRH in fragile and conflict-affected states exposes the limitations of this approach. Where the reach of the state, at least in terms of service provision, is limited and where the classifications commonly used in HRH do not apply, available data are inevitably compromised. Furthermore, service provision is fragmented, and the categories of public and private providers are blurred. In this context, a full understanding of HRH is not helped by the use of the narrow state-centric view [7].

### The six fragile and conflict-affected countries

Each of the states examined has a different history, yet they share several commonalities. In each, despite prolonged international efforts, state building has largely failed. With the state elusive, in disarray and frequently mistrusted, its capacity to afford citizens protection from conflict and to provide the full range of essential services is tenuous [5]. Each of the countries examined has experienced conflict or political instability and a vicious cycle of insecurity, weak rule of law, a flourishing illegal economy, extreme deprivation and, consequently, deleterious health outcomes. With the exception of the Occupied Palestinian Territories, key health indicators (such as life expectancy, neonatal and infant mortality and maternal mortality) are dire compared to regional averages. Table 1 provides an overview of basic health statistics in each of the case studies.

**Table 1:** **Basic health indicators for each case study**

	**Life expectancy and mortality**				**Age-standardised mortality rates by cause (per 100 000 population)**			
	**Life expectancy at birth (years)**	**Neonatal MR**	**U5 MR**	**Adult MR**	**Communicable disease**	**Non-communicable disease**	**Injuries**	**Measles immunisation among 1-year-olds (%)**
Afghanistan^a^	48	45	149	M: 440; F: 352	713	1 117	149	62
CAR^a^	48	42	159	M: 461; F: 470	1 060	870	151	62
DRC^a^	49	46	170	M: 442; F: 331	932	837	155	68
Haiti^a^	62	27	165	M: 278; F: 227	545	697	52	59
Palestine^b^	73	13	23	N/A	N/A	N/A	N/A	N/A
Somalia^a^	51	52	180	M: 382; F: 350	736	967	199	46

^a^Data retrieved from WHO, 2012, World Health Statistics; ^b^data retrieved from UNICEF (2013). At a glance: state of Palestine. Retrieved 11 August 2014 from http://www.unicef.org/infobycountry/oPt_statistics.html.

### Study design

This examination of HRH is one component of a larger study examining the provision of health services in six fragile and conflict-affected states. The project was funded by the Danish Ministry of Foreign Affairs, and implemented through the Australian Centre for International and Tropical Health (ACITH), at the University of Queensland, using a research team of academics and independent consultants experienced in, or at least familiar with, health systems analysis in distressed contexts. The overall purpose of the project was to provide greater understanding of the provision of health services in such environments and to explore how health systems react, adapt and evolve in response to total or partial state failure. The findings of other components of the larger study have been published in peer-reviewed publications and in detailed country reports [7-11].

The study followed a case study design [12], using primarily qualitative methods: documentary analysis and key informant interviews between 2010 and 2012. The qualitative methods allowed for an exploration of how and in what ways health systems evolve, or might be evolving, in response to state fragility. This design proved particularly useful in addressing the research purpose because of its ability to describe, explore and offer explanations for the phenomena being studied [13-18]. The documentary analysis used available peer-reviewed articles, books and “grey” literature—unpublished research and evaluations and reviews from multilateral and bilateral donors and non-government organisations. The available literature outside the health field was studied in some detail, to gain insights about the influence of such turbulent contexts on the respective healthcare arenas. A common thematic guide, developed by the research team and informed by the documentary analysis, mapped themes related to health service provision and the impacts of social, economic and political disruption on health services and HRH. A semi-structured question guide was developed for the key informant interviews. The use of the shared thematic guide ensured parallel coverage of key issues between studies, while individual researchers were able to respond with flexibility to issues raised by respondents [19].

In each of the contexts examined, key informant interviews were undertaken with participants identified from ministry of health (MoH) organograms, listings of donors, non-government organisations (NGOs) and independent professionals, based on their health systems engagement and experience. Non-health informants were also interviewed. Additional key informants were identified by recommendation and referral from the initial round of interviews [14,16,20,21]. In Haiti, respondents were selected based on a review of the Ministry of Public Health and Population (MSPP) organogram and the coordination list for health organisations. These respondents suggested other informants including the Cuban Brigade and representatives of the Département at Cap Haïtien. In Afghanistan, respondents included representatives of the Ministry of Public Health, donors, NGOs, other humanitarian organisations and UN agencies. In CAR, five of the seven accessible prefectures outside the capital Bangui were visited. In Somalia and the Occupied Palestinian Territories, informants were interviewed about specific issues related to health and HRH. For reasons of insecurity, interviews with informants in the health sector in Somalia were conducted primarily in Nairobi, headquarters to most donor programmes in Somalia.

The researchers sought to achieve representative cross-section across state, private for-profit and not-for-profit actors, local and international informants, central agencies and provinces, practitioners and academics. The total number of interviews for each study was primarily determined by the numbers of individuals identified in the field as being knowledgeable about the healthcare arena or other relevant issues and the feasibility of access. Specific attention was given to the volume of available literature when planning interview schedules. For Somalia and the Occupied Palestinian Territories, both well documented, the focus was on corroborating—or challenging—existing evidence. The numerous interviews undertaken in the CAR was in compensation for the paucity of studies, with additional interviews in the CAR reflecting extended access to five prefectures. The total number of interviews was as follows: CAR *n* = 74, Haiti *n* = 45, Afghanistan *n* = 41, DR Congo *n* = 32, the Occupied Palestinian Territories *n* = 30 and Somalia *n* = 25. The corroboration of data from informants confirmed that saturation had been reached within this selection. Key informant interviews were combined with site visits to at least one provincial centre in each of the states examined, with the exception of Somalia. This allowed researchers to identify and explore discrepancies between official discourse and the reality observed on the ground. In relation to Afghanistan, the Occupied Palestinian Territories and Somalia, the analysis built on previous work done by the researchers on their healthcare arenas and facilitated by the network of contacts established over years of protracted engagement.

Identified key informants were contacted by phone or face-to-face, individually briefed on the purpose of the research and interviewed at their work premises or at an agreed location, with notes maintained. Interviews were undertaken in English or French depending on the interviewees’ preference, with all researchers competent in both languages. All interviews were undertaken following reading of the project information sheet, outlining the purpose of the research and the provisions for confidentiality, and emphasised the right to withdraw at any time and confirming informed oral consent. Table 2 summarises the number of respondents by category and location.

**Table 2:** **Summary of participants by category and locations**

**Participant category**	**Locations**							
Afghanistan location	Kabul			Herat Province				
Academic body	2							
Afghan hospital	1			1				
Afghan NGO	3			2				
Bilateral organisations	1							
Government	14							
International NGO	8			3				
Other	1							
Multilateral organisations	6							
Central African Republic location	Bangui	Bambari	Bimbo	Bobele	Berberati	Ippy	Kaga-Bandoro	Mbaiki
Ministry Public Health	5		1					1
National organisations	20		1					2
Academic body	4	1						
Bilateral organisations	1							
CAR health professional	7	8		14	6	2		
CAR consultant								
Multilateral organisation	7						1	
Bilateral organisation	3							
International NGO	14	4			1			
International consultant	4							
Other	2							
Democratic Republic of Congo location	Kinshasa		North Kivu	Goma		Antwerp		Geneva
Ministry Public Health	7							
National organisations	6							
Academic body	3							
Bilateral organisations								
DRC health professional	1		1					
DRC consultant								
Multilateral organisation	6			2				2
Bilateral organisation	4							1
International NGO	3							
International consultant	3							
Haiti location	Port-au-Prince					Cap Haïtien		
MSPP	5					1		
Academic (Haïti)	4							
Haïtian hospital	2					1		
Haïtian NGO	7							
Haïtian consultant	2							
Cuban Brigade						2		
Multilateral organisation	8					1		
Bilateral organisation	2							
International NGO	7					1		
Palestine location	Jerusalem		Nablus	Ramallah		Gaza		Abroad
Palestinian academics				2		1		
International academic								1
International consultants	2							
Bilateral organisations	3							
Palestinian National Authority				1				
Ministry of Health			4			3		
Multilateral organisations	1		1			2		
Palestinian NGOs				3				
International NGOs				1				2
Folk healer			1					
Private firm				1				
Somalia location	Mogadishu		Somalia	Nairobi		Abroad		
Academics	1			1		1		
International consultants				2		4		
Bilateral organisations								
Somali health professional	1		3					
Multilateral organisations				7		1		
Somali NGOs				1				
International NGOs			2	5				

### Data analysis

Researchers shared a common process of analysis for the preparation of each of the case studies. Thematic analysis was undertaken manually and corroborated with the research team members. The common thematic guide, developed before the data collection, provided the initial themes for analysis, with data from the documentary analysis and key informant interviews integrated in the findings. The analysis was guided by the broader project research questions, with a report written for each of the six contexts examined. Subsequently, the data were reanalysed with a focus on those themes relating to HRH. In the draft report writing stages, the researchers integrated the qualitative findings with the literature into a primary report for each location studied. Initial drafts of the six different reports were shared with colleagues regarded as able to provide relevant feedback and insights. This was followed by a review undertaken by the whole research team. The final reports are available from the ACITH website^a^.

Triangulation of qualitative case study information involves collecting data from different locations and cross-checking their consistency from various sources collected at different times [12-15,22,23]. In this study, data triangulation was achieved by including a wide range of documents in the review, interviewing international and national healthcare experts, presenting preliminary findings to colleagues experienced in each study location and undertaking an internal review processes within the research team. In the case of Afghanistan and Somalia, subsequent professional visits by the researchers have allowed confirmation of findings. These processes helped to improve our understanding of the various reasons for differences and similarities in the data and identify areas for further exploration.

Ethics approval for the research was obtained from the University of Queensland Research Ethics Committee. In Haiti, the research protocol was shared with the MSPP, and they were informed of the ethics approval. In the CAR, the Ministry of Health was informed of the study and gave formal approval for its health facility staff to participate. In the DR Congo and Afghanistan, the proposal was provided to the respective health ministries.

### Limitations

Some of the limitations of this study relate to undertaking research in extremely distressed environments. Security considerations for the researchers were significant: the reach of the research was extended considerably by members of the team who, as independent consultants, were not constrained by University of Queensland travel restrictions. Each study except Somalia included both central and provincial visits, though the majority of informants—both state and non-state, with the exception of the Occupied Palestinian Territories—were concentrated in the capital and this inevitable capital city bias is an historical artefact. With additional time and then-relative security, the fieldwork in the CAR covered significantly more provincial centres and proportionately larger number of interviews reflecting regional differences. Another potential limitation is that the data for this study has come from arguably extreme cases of state fragility and may not have widespread applicability. On the other hand, the weakness or absence of the state allowed a more in-depth, nuanced approach and identification of patterns which, while also present to varying degrees in less distressed settings, are typically harder to uncover or understand.

In this research, our intent was to refocus the analytical lens to the HRH system as a whole. Our research uncovered the extraordinary health services activity beyond the reach of the state, but time, resource constraints and limited available data HRH (and many inaccuracies in available data) prevented a comprehensive in-depth analysis of the active workforce including the formal, informal, private, nongovernmental and faith-based providers, as well as an examination of factors that determine the demand for HRH and the dynamics of the formal and informal labour market. Another limitation of the study was the failure to secure demand-related data (in other words, client perceptions) in most settings, leaving this for further research. Finally, while the approach allowed the collection of rich qualitative information, the study was also constrained by the inconsistent availability of information in each of the study locations, resulting in uneven insights in relation to the different HRH sectors. Lack of documentation hindered the appreciation of trends and the direction of movement of developments [24].

Accepting the variety of patterns spontaneously occurring in a long-suffering healthcare arena calls for appropriate analytical tools, which supersede the definitions and indicators applied in stable, better organised and resourced contexts [25]. For instance, the much referenced “international ratios” of professionals-to-inhabitants are meaningless in situations where most workers do not hold a recognised qualification, those who do so demonstrate questionable skills, and the population concerned is of unknown size and very mobile. The same fuzziness applies to health facilities, with atypical services—featuring a limited range of services or staff with qualifications that do not conform to usual vocational classifications—vastly outnumbering standardised health facilities. Relying on conventional staffing patterns is therefore similarly misleading. A third example may relate to the proportion of deliveries attended by skilled workers. Without a direct assessment of the actual skills demonstrated during the delivery, such indicator is obviously devoid of significance. Equally, one clinician’s “outpatient consultation” (a common measure of workload) cannot meaningfully be compared with another’s given the absence in quality control that regular supervision affords, itself often entirely absent. Despite these limitations and selecting the case studies for maximum diversity, the study identifies shared important commonalities across the six cases.

### Findings

In each of the study’s six states, severe deficiencies in HRH information systems, misdistribution of HRH and weak regulation and oversight of HRH by the state were observed. In each case, it became apparent that the readily available data focussed on formally public health services. Information about the actual practice adopted by public employees, in fact dominantly privatised, was sorely inadequate. Official data were limited, frequently irregular, often palpably incorrect and addressed only a small proportion of human resources [25]. Despite this, the research provided important qualitative insights into HRH in these settings, pointing to the implications for an actual active workforce concealed through the blurred boundaries of double employment, un-integrated not-for-profit providers and the unregulated for-profit private operators, where the bulk of health services is provided. In each of the healthcare arenas investigated, the problems faced by HRH are consequences of a combination of economic and governance functions (or dysfunctions). As a result, service provision defaults into patterns driven by financial considerations and, from there, the easy commodification of health services.

### The uncertain size of the health workforce

In each of the six states examined, an accurate estimate of the size of the health workforce was unavailable and what did exist looked incomplete. In most cases, health workforce data were reliant on personnel records, covering only salaried staff in the centralised public sector, frequently referring to outdated enrolments. Non-salaried personnel working within state facilities were not included. In the CAR, an unknown but sizeable percentage of the workforce was reported (and seen) to be entirely “off-ledger”. Absenteeism among those “on-ledger” was reported as common and unchecked and may be for reasons both authorised and unauthorised.

In the DR Congo, informants conceded that the total number of health workers was not known with any certainty. In the popular mind, every health worker, even auxiliary staff member, is a *nganga*, or “doctor” (and every health facility is a nganga house). In Haiti, there were considerable difficulties in gaining an overview of HRH, their capacity and their gaps due to an inadequate health workforce information system, currently under revision. Within each ministry, the management of human resources (HR) is under-developed and given a lower rank.

The disconnection between modest service needs (constrained by financial factors) and vigorous supply of health professionals was recognisable in all settings. Despite the underlying uncertainties about the true size of each workforce, time series pointed unambiguously towards a sustained growth, particularly in relation to the DR Congo and the Occupied Palestinian Territories, both endowed with large health training capacity. This trend has been recognised also in other healthcare arenas, such as in Angola [26]. Counter-intuitively, an expanding workforce looks like a recurring feature of the diminished state, which relinquishes its grip on supply, employment and professional practice. The profit prospects of healthcare activities, within a suffering domestic economy, attract both workers and entrepreneurs.

An accurate inventory of Palestinian health professionals and estimates of outward migration were likewise not available. In Afghanistan, the portion of trained community midwives going into practice in the public sector could not be established; many, however, were reported to marry and stop practising, and the use of midwifery training as a bridge to higher academic pursuits was commonly reported [27]. In Somalia, compilations of HRH-related data quickly revealed inadequacies, grossly diverging data, inconsistencies in classification and limited coverage.

### A mal-distributed workforce

In each of the study locations, a number of respondents questioned the reliability of the HRH data. Despite the paucity of accurate quantitative data, mal-distributions by gender, by professional categories and by urban/rural allocation of health workers were recognisable in all of the settings examined. In the CAR, for example, HRH distribution across the prefectures was reported to be markedly uneven. Successive waves of conflict had contributed to the large-scale flight of qualified staff from almost every facility in the entire north and north-eastern prefectures. In those areas, most of the available formal HRH were employees of not-for-profit providers allocated according to organisational discretion, an example of organisations on the ground making resourcing decisions rather than far-away ministries of health who do not have the information or the capacity to deploy staff productively. Isolated multi-disciplinary teams existed in the rural south-east due to the intervention of an international NGO. The far north-eastern prefecture was reportedly almost entirely devoid of health providers. Even where an international NGO provided services, they were available for Sudanese refugees only.

In Afghanistan, trained midwives were unwilling to fill rural vacancies, preferring unemployment to perceived undesirable rural appointments. In the absence of female doctors, female patients were reported to consult with Afghani community midwives for a range of conditions rather than see male doctors [23]. The *de facto* privatised public health services, with not‐enrolled health personnel paid only by user fees, were reported to contribute to overstaffing in areas where people could pay, while there was understaffing in poor areas. In Haiti, 83% of specialist doctors were practising in the capital, Port-au-Prince [28]. In some of the states included in the study, violence against health workers, for example, Taliban threats to female nurses and midwives in Afghanistan, contributed to the further distortion of the health workforce. On the other hand, in some areas, humanitarian agencies provided superior health services in conflict-affected areas whereas stable by poor communities with equally poor health outcomes, continued to be underserved.

The centralised nature of the HR system in Haiti also caused unnecessary delays in replacing medical staff [29]. The departmental director for the MSPP, for example, had no authority to hire and fire. This led, for instance, to anomalies where a clinic director reportedly had three drivers on the payroll without a car at his disposal or to a hospital director being allocated five cleaners instead of the requested five midwives. The production of graduates was poorly linked with service needs. The uncertainty around subsequent employment by the state or access to specialist training, coupled with the opportunities for work in the international marketplace, led to high levels of migration of medical graduates in Haiti. Lack of regulation of educational institutions also resulted in disparities in the output of different professional categories. Medical training was preferred over other categories of health professionals, leaving gaps in the production of nurses and midwives, for example, and with training of physiotherapists only initiated following the 2010 earthquake.

In Somalia, the health workforce had expanded organically over the years with no link between workforce requirements and professional training. This was also observed by the practice of absorbing not only holders of recognised qualifications but also self-appointed health workers, as well as “volunteers” who sometimes charged a fee for service in the absence of salaried workers. As a result, protracted unemployment in the salaried public health sector was a common occurrence for health graduates [30]. To redress this, recurrent ministerial instructions were issued in Somaliland to enrol new batches of health professionals, regardless of service needs or actual utilisation. Professionals from the violent central-southern region migrated to peaceful Somaliland. The inadequacies of country-wide inventories cannot be resolved by more thorough counts, which would in any case become quickly outdated. Stronger insights can be obtained from local surveys, which were available in some cases, such as in the eastern DR Congo, Somalia or the Occupied Palestinian Territories.

### Fending for themselves

Those in the informal subsector fend for themselves by definition: those in the public subsector do so by circumstance. A perceived inadequacy of salaries, the late payment of those salaries (in CAR, sometimes for an entire year, only to be written off by presidential decree) and poor working conditions encouraged staff to work privately outside their official job (but sometimes within their working week), work privately inside their official job, unofficially charging for notionally free services or taking up other jobs altogether [31]. Ineffective oversight by line-managers—themselves feeling inadequately paid and poorly rewarded—only facilitated these self-preservation efforts. In Haiti, some doctors on half-time contracts were present only once a week. Others were present only once a month—to receive the pay cheque, leaving the work to interns [29]. In the CAR, all levels of health workers in state facilities essentially functioned as stand‐alone entrepreneurs with minimum overheads. For example, specialists and qualified midwives operated private clinics within the publicly owned hospital infrastructure. An important reason for professionals in the CAR, Somalia and elsewhere to remain employed in the public subsector is that such an appointment confers status, reputation, credibility and opportunities which increase market profile—and market share—in the private subsector. In CAR, qualified health workers not on the state payroll openly worked within public facilities on a formally sanctioned fee-for-service arrangement, with revenues divided at the end of the month;

Similarly, in the DR Congo, numerous health workers present in public health units were not on the government payroll. In Katanga in 2007, for instance, it was estimated that, out of a total of 6 800 health workers, only about 3 200 were officially employed [32]. This contributed to the *de facto* privatisation of nominally public health services [32]. In Somalia, private practice generates most of the income for a majority of health professionals. A study in the Occupied Palestinian Territories found three quarters of government employees interviewed were also working at NGO or private facilities [33].

In Afghanistan, interviewees indicated that the HR boundaries between NGOs and the MoPH are fuzzy; managerial staff members switch between both during their career. With salaries for health workers in NGOs at least 50% higher than government salaries, moving to NGOs, UN organisations, donors and coalition forces was reported as commonplace. Those less in demand, such as community health workers, were not afforded similar opportunities to move, creating a rift between those who were paid by some NGOs (against official policy) and those who were not. In Haiti, disparities between salaries and conditions in the public and private sectors were reported to lead to apparent paradoxes: a cleaner working in a cholera treatment centre run by an NGO, for example, could earn more than a nurse-assistant of the public clinic to which the NGO centre was attached. There is only an apparent paradox: the latter health professionals would have more opportunities than the former to supplement their income through charging patients for their services.

There is a strong correlation between the presence of health workers at any given service delivery point and the availability of medicines: sales of which may constitute the bulk of actual earnings. Where official supplies are inadequate or absent, some health workers buy medicines in the open market and sell them, with a mark-up, to their captive patients [34]. Expensive products are often prescribed for their higher returns rather than their therapeutical value.

### The unregulated production of human resources for health

While no figures were available, in most of the six healthcare arenas examined, the training of health professionals was an apparently profitable business judging by its attractiveness to entrepreneurs. With much upward pressure on quantity to expand class numbers—often fuelled by the students’ benevolent relatives in the diaspora—and negligible downward pressure on quality through absent or nominal regulation, the training of health staff combined high profit margins with low start-up costs. This resulted in an unplanned proliferation of private, for-profit, heterogeneous training outlets. Pre-vocational education was uneven and suboptimal, often inappropriate in orientation with a focus on professional categories with larger earning potential, such as doctors and pharmacists. In lock-step, the proliferation of heterogeneous examining bodies, leading to similarly uneven competence and certification, matched the expansion of the training outlets.

According to informants in Afghanistan, a diploma, far from being a guarantee of a satisfactory level of knowledge and skills, was rather merely an automatically awarded certificate delivered upon completion of a course. The hierarchical nature of Afghani society was reported to constrain on-the-job training. In a provincial hospital, for instance, “doctors took questions during teaching rounds as a personal attack”. The low level of general education resulting from protracted turmoil also limited professional development and was reported to be particularly influential in the failure to redress the male/female health worker imbalance. Persistent shortfalls in recruited female health workers in Afghanistan, despite a doubling of the hardship allowance for female staff, posed a major obstacle for access of women to health care. Added to this, misconceived attempts to raise nursing and midwifery standards contributed to gender disparities: candidates were required to have completed secondary education—a situation denying entry for many rural women.

In the DR Congo, the proliferation of medical schools and training institutions resulted in an over-supply of health workers, many with substandard healthcare qualifications. According to the Ministry of Public Health, between 1998 and 2008, the number of faculties of medicine had increased more than tenfold from 3 to 39. In the same period, health training schools for allied health cadres almost doubled from 308 to 578. A fifth of medical schools were reported to not meet the required standards, and a quarter had no associated teaching hospital. Nevertheless, these same institutions were allowed to continue to operate, every year providing more than 2 000 new doctors—of whatever standard—and more than 4 000 new nurses [35].

In Somalia, local entrepreneurs, funded by external sources, donations from local wealthy people, the diaspora and student fees established training institutions for health professionals. Informants noted that in many locations across the former Republic of Somalia the healthcare industry ranks among the top employers, and enrolling into health training programmes represents an appealing option for school leavers. In Haiti, the absence of a training facility accreditation system and the use of curricula not matched to the needs of the Haitian health system negatively impacted on health staff performance [28]. In private nursing schools, the curriculum was described as “sclerotic and teaching methods 50 years back”. Several practising physicians complained about the decline of standards of medical training. Further, the HRH training industry was reported to have grown dramatically, apparently saturating the market. Given, however, that graduates can use their qualifications to migrate, it seems unlikely that the domestic market for health professionals will remain saturated for long as more graduates migrate. Educators, for example, suggested that approximately 80% of doctors migrate on graduation. In the case of the Occupied Palestinian Territories, training geared to the international job market was flourishing. Many healthcare staff benefitted from scholarships abroad, resulting in diversity in standards and quality of graduates.

In the CAR, many of medical doctors trained in a 7‐year course at the University of Bangui were thought to have migrated. On the other hand, one informant explained that “there are many unemployed health professionals” still in-country, but in the absence of accessible records, obtaining even a rough assessment was challenging The training of *secouristes* is a core mission of all national Red Cross societies, and the CAR branch annually trains two batches of 50 at each of five sites in 3-month courses or around 500 each year. This basic training offers a foot on the health worker ladder: half of all *mini-pharma* informants claimed to have had Red Cross training, and *secouristes* make up most of the volunteers who staff village health posts. In the DR Congo, approximately 11 000 medical doctors were registered; only about 5 000 of whom are estimated to be working in the public sector. Presumably more than half of graduate doctors migrated, took up other jobs or practised privately.

In Somalia, health workers could be placed on a continuum from the “reasonably qualified” (maybe with foreign qualifications) to the “indubitably unqualified”, with most workers falling in between. There was no automatic correspondence between qualification and competence: some “qualified staff” remaining inside the formal system with a quasi-academic certificate were incompetent while some very competent staff (having continued to deliver services seen as acceptable by customers through decades of disruption) remained unqualified.

In the six healthcare arenas examined, professional associations were found to exist to differing degrees, sometimes if only to demand a tax from new entrants to the market but rarely, if ever, to provide professional support.

## Discussion

The findings pose a question: given the demonstrable limitations, could a formal health system be said to exist in any of the six examined contexts? If the criterion for a health system is the existence of a dynamic mix of health services and a range of public, private, formal and informal providers, then the answer may be “yes” [11,36]. What this study underlines is the observation that despite the absence—or in some cases, withdrawal of the state—the healthcare arena and the HRH space specifically is not a vacuum. This finding distinguishes this research. Beyond the reach of state services, health provision is crowded with a range of private actors of varying competence, public servants turned health entrepreneurs, and a diverse richness of initiatives, shaped by the unregulated commodification of health care. While the specific findings from the six cases cannot be generalised, the privatisation, commoditisation and range of formal and informal providers offering healthcare services has been observed in other weakly governed contexts [37,38]. Further, despite differing histories, similar trajectories were found across the six case studies.

In each of the six healthcare arenas, formal coordination and regulation systems were either absent, simply not applied or abused to financially exploit patients and lower level staff [5]. There was minimal skill development or evidence-based practice. As a result, HRH was governed largely by informal rules and shaped by socio-economic and political determinants, local culture and local value systems. With *de facto* stewardship in the hands of the market, market forces and profit-based motivation determined the range and quality of available health professional training, the distribution of services and performance. Unregulated training institutions, financed by student fees, contributed to questionable training of questionable quality and the (over-) production of often under-skilled health cadres with dubious qualifications. Further, while training for medical doctors proliferated, the number of trained nurses and midwives trailed. Poor quality training and the commodification of health also meant healthcare workers rarely acted as perfect agents for patients’ health. Similarly, leaving the distribution of the workforce to the market results in disparities in service provision. It exacerbates the existing inequalities often observed in under-governed health systems and can result in catastrophic events for families [27,38-42]. Elsewhere, the mix of the requirement to pay for health services and products, limited ability to pay and the lack of health insurance have been shown to result in catastrophic payment while at the same time payments provide important revenue at the facility level especially when reimbursements from the state are frequently delayed [43-45].

Strengthening the role of any putative state to take on its enabling role and establish governance mechanisms will be a protracted, incremental exercise, one likely to take decades to reach a basic level of functioning [46-49], with no guarantee that it will happen [11]. In each of the case studies, the fragility of governance structures is not new; rather, it is chronic and stubborn to international interventions. Even if a state-based governance structure registers progress, it will relate only to a modest portion of the healthcare market. And publicly employed health workers will remain subject to market pressures determined outside the public sphere. Thus, a focus on building the capacity of the state on its own is unlikely to result in tangible improvements. Our analysis suggests that conceptualising the HRH arena at the national level and focussing on the public health sector is, to an extent, misleading and provides a parody of reality. A more useful conceptualisation may be to look at local healthcare provision arrangements in their diversity including formal and informal as well as private and public elements [50]. This would provide a more holistic understanding of the HRH resources available to enhance the quality of health care and enable a piecing together of a system from the reality of what is, rather than on the illusion of what is not, drawn from a focus on idealised patterns [5,38,40,50-52]. This more comprehensive analysis of HRH will allow for the development of strategies to support and develop existing resources, promoting adaptive responses to existing dynamics [5].

The challenge, then, for the international community is to find ways to effectively engage with the system as a whole [53], essentially looking afresh at the healthcare arena and searching out all actors, working out new ways to engage with them. In other words, it means recognising the state as merely one actor and finding ways to at first recognise and then encompass all others. It means that donors and international organisations need to learn to work in different ways and extend their analysis beyond the notion of a state-led health system, accepting that in fragile and conflict-affected environments the healthcare space is fuzzy, fluid and fragmented. It also means accepting risk as an intrinsic part of change which provides opportunity for learning and adaptation. To minimise risk, interventions need to be incremental, combining strategic intent and structured intervention, while at the same time building in flexibility for adaptation to take into account emergence and complexity [54]. Such an approach will also require a highly flexible financing system that can support local providers and essential service delivery in the immediate term while building capacity in the longer term [42].

Realising that most health workers who lack either formal qualifications or actual skills will keep practising even if officially forbidden to do so points to the measures needed to resuscitate a moribund workforce. In particular, new ways need to be found to manage the workforce in four areas: workforce production, distribution, performance and coordination and regulation [5,39].

1. In light of the large number of active health workers, managing them, upgrading their skills and encouraging positive behaviours should take precedence over training new ones in most contexts. Deployment, fulfilled tasks, workloads, terms of employment, incentives and career prospects need to be better understood if adequate measures have to be introduced [39,55,56].
2. An independent and voluntary certification process, along the lines experimented in Cambodia and Afghanistan, should be instituted [57]. Precious insights would be gained about the competence of practising health workers, their most glaring skill gaps and the adequacy of existing categories and, henceforth, of their retraining needs [32,56]. Importantly, more research is needed on understanding which incentives would make an untrained medicine vendor, for example, behave differently: promoting health rather than promoting what the consumer sees as health, in the context of no formal regulations and no likelihood of their execution [35]. Ensuring appropriate and uniform good quality training is particularly challenging, especially given many training providers are privately owned [49,56], and further research is needed to better understand how to align different actors’ motivational interests [53].
3. The accreditation of health training institutions deserves an investment, with the removal of automatic expectations of enrolment in the state workforce, if the oversupply of incompetent health workers has to be reined in. While more attention needs to be given to strengthening the capacity of the state to undertake its stewardship role in developing and enforcing licensing and accreditation systems [5] given the feebleness of health authorities, in the interim, a voluntary mechanism could be offered. If the state is mistrusted or contested, an international body could be assigned this role. In an open healthcare market, a reputable accreditation system may induce training institutions to abide to norms otherwise ignored [5]. Market forces should then mould their efforts, and further research is needed to understand how to develop the capacity of consumers to demand appropriate and quality services [49,58]. Further, without linking production to deployment, accreditation of health training institutions on its own is likely to bring limited benefits if not linked to deployment and retention [5].
4. Normative planning, whereby the health workforce is projected to expand according to absolute needs, should be abandoned in favour of contextualised criteria taking resource and capacity constraints into due account [56]. Indeed, different service models and healthcare networks have different HR requirements, whatever the served population is. Forecasted financing levels offer a much better guidance to HRH development than international norms (themselves the result of averaging vastly diverse situations) [59,60]
5. Coordination, monitoring, evaluation and regulation are vital in the strengthening of HRH and should include a range of stakeholders [5]. Where the capacity of the state to coordinate is weak, local coordination mechanisms can be facilitated by identifying local governance structures. Given changes in one system, for example, the HRH system is likely to impact on other parts of the broader health system; systematic coordination is also needed between the HRH system and other subsystems of the overall health system [44]. McPake et al. for example have demonstrated the interconnectedness between the HRH systems and the financing system [44]. Similarly, Bertone and colleagues observed in Sierra Leone that broader health financing reform, while not specifically focused on HRH, had a substantial impact on the HRH reform process [61].

To conclude, our research highlights the need to recognise that public health authorities are only one of many actors in the health field and that reversal of fragility is a long-term commitment and demands a form of engagement which deliberately aims to harness the strengths of the diverse actors that provide health services. It requires a shift from a reductionist, predictable and linear view of health systems to one which acknowledges the complex character of health care in severely distressed environments. Crucially, improving HRH in fragile and conflict-affected areas requires researchers and policy makers to embrace the inclusive WHO definition of the health system. As compelling and easy as it is to focus on the public sector, this provides a caricature of reality. A more holistic scrutiny of the sections composing the healthcare arena and of their interactions will provide a more coherent understanding of the HRH dynamics in fragile and conflict-affected states. Importantly, it will allow identification of windows of opportunity for novel and experimental interventions. The approach will not be risk-free and demands tolerance for ambiguity and uncertainty around the likely effectiveness of interventions. The dividends will be realised, however, through the harnessing of the HRH active on the ground and the practices they adopt to survive. Meaningful interventions oriented to public goods must cover the whole market and not only its formal public arm. The next research agenda should aim at exploring the complex and dynamic structures of supply and demand in the HRH market in under-governed healthcare arenas, in order to identify existing or emergent systems of governance and to work with them to improve population health outcomes.

## Endnote

^a^ACITH (2012) http://www.sph.uq.edu.au/acith.
